# RASSF Family Proteins

**DOI:** 10.1155/2012/938916

**Published:** 2012-12-10

**Authors:** Geoffrey J. Clark, Shairaz Baksh, Farida Latif, Dae-Sik Lim

**Affiliations:** ^1^JG Brown Cancer Center, University of Louisville, 417 CTR Building, 505 S. Hancock Street, Louisville, KY 40202, USA; ^2^Division of Hematology/Oncology, Department of Pediatrics, Faculty of Medicine and Dentistry, University of Alberta, 3-055 Katz Group Centre for Pharmacy and Health Research, 113 Street 87 Avenue, Edmonton, AB, Canada T6G 2E1; ^3^School of Clinical and Experimental Medicine, College of Medical and Dental Sciences, University of Birmingham, Edgbaston, Birmingham B15 2TT, UK; ^4^National Creative Research Initiatives Center for Cell Division and Differentiation, Department of Biological Sciences, Korea Advanced Institute of Science and Technology (KAIST), 373-1 Guseoung-D, Yuseong-G, Daejeon 305-701, Republic of Korea

## 1. RASSF Family Proteins

The Ras-association domain family (RASSF) proteins are tumor suppressor proteins whose importance to the development of cancer has become increasingly apparent over the last 12 years. While possessing no enzymatic activity, they appear to function as scaffolding molecules to regulate the activity of a surprisingly broad array of effectors. They are implicated in the regulation of a diverse range of biological functions including apoptosis, autophagy, cell cycle control, microtubule dynamics, and DNA repair. In addition, they are thought to be one of the regulators of the Hippo pathway, the newly emerging tumor suppressor pathway evolutionarily conserved between *Drosophila *and mammals.

Typically, inactivation of RASSF genes in cancer involves epigenetic silencing of their promoters. Indeed, the RASSF1A promoter appears to be the most frequently inactivated promoter yet detected in human tumors. As PCR-based assays may be used to detect specific promoter methylation in body fluids with exquisite sensitivity, it may be possible to use the epigenetic inactivation of RASSF genes as prognostic/diagnostic markers. In this issue, inactivation of RASSF genes in hepatocellular carcinoma and bladder cancer is considered by D. F. Calvisi et al. and W. Meng et al. as examples.

 The RASSF1A gene may be unique in the family as it also found to be frequently mutated in cancer cells. These mutants may provide useful tools for structure/function studies. Moreover, RASSF1A exhibits a polymorphism which is common in Caucasians and is associated with an enhanced risk of cancer. M. Gordon et al. include a comprehensive review of the current knowledge regarding RASSF1A mutants and polymorphisms.

RASSF1A is the best-characterized member of the RASSF family and most of our knowledge regarding the biological function of these proteins derives from studies with RASSF1A. It can modulate at least two major apoptotic signaling pathways. One via a modulator of apoptosis (MOAP-1)/BAX pathway, and the other via the intriguing and perplexingly complex hippo pathway. The interaction with the Hippo pathway may occur at multiple levels and may permit RASSF proteins to modulate cell cycle effects as well as apoptosis. J. Law et al. describe our current understanding of the MOAP-1 pathway while F. Fausti et al. and A. M. Richter et al. elaborate on the role of the Hippo and RASSF/Hippo connection in regulation of several aspects of biology.

 RASSF1A may also impact the cell cycle and genetic stability by the modulation of microtubule dynamics. RASSF1A binds several microtubule-associated proteins (MAPs) directly and may use these to modulate microtubule dynamics essential to motility and spindle formation. This function may have therapeutic ramifications by influencing the sensitivity of tumor cells to microtubule-targeting cancer therapeutic agents such as Taxol, as described here by S. Kassler et al. A key role for RASSF1A has also been identified in modulating the DNA damage response. These aspects of RASSF1A are considered by S. F. Scrace and E. O'Neill. Thus again, the RASSF1A status of a cell may influence the response to a therapeutic intervention, in this case, DNA damaging agents. A similar situation arises with the family member RASSF2, as described by J. Clark et al. The potential for epigenetic therapy to reverse the loss of function of RASSF genes is plausible and may enhance therapeutic approaches.

 Although RASSF proteins are primarily regarded as tumor suppressors, there is now strong evidence that they may also play a key role in cardiac function. In particular, RASSF1A is important for hippo pathway driven cardiac hypertrophy responses. D. P. Del Re and J. Sadoshima add a review of the cardiac role of RASSF1A to complete the Special Issue.

 The field has been catalyzed by an inaugural international RASSF symposium in 2009 in Banff, AB, Canada and a second meeting in Oxford, England in 2011. A third meeting is being planned for 2013 (RASSF Symposia Information at http://rassfsymposia.com/). These meetings brought together scientists and clinicians from all around the globe to share information and debate results. In the coming years, we anticipate more revelations demonstrating the biological importance of RASSF family proteins in human development and disease.

*Geoffrey J. Clark* *Geoffrey J. Clark* *Shairaz Baksh* *Shairaz Baksh* *Farida Latif* *Farida Latif* *Dae-Sik Lim* *Dae-Sik Lim*

